# The spike of SARS-CoV-2 promotes metabolic rewiring in hepatocytes

**DOI:** 10.1038/s42003-022-03789-9

**Published:** 2022-08-17

**Authors:** Maria Mercado-Gómez, Endika Prieto-Fernández, Naroa Goikoetxea-Usandizaga, Laura Vila-Vecilla, Mikel Azkargorta, Miren Bravo, Marina Serrano-Maciá, Leire Egia-Mendikute, Rubén Rodríguez-Agudo, Sofia Lachiondo-Ortega, So Young Lee, Alvaro Eguileor Giné, Clàudia Gil-Pitarch, Irene González-Recio, Jorge Simón, Petar Petrov, Ramiro Jover, Luis Alfonso Martínez-Cruz, June Ereño-Orbea, Teresa Cardoso Delgado, Felix Elortza, Jesús Jiménez-Barbero, Ruben Nogueiras, Vincent Prevot, Asis Palazon, María L. Martínez-Chantar

**Affiliations:** 1 Liver Disease Lab, CIC bioGUNE, Basque Research and Technology Alliance (BRTA), 48160 Derio, Bizkaia Spain; 2 Cancer Immunology and Immunotherapy Lab, CIC bioGUNE, Basque Research and Technology Alliance (BRTA), 48160 Derio, Bizkaia Spain; 3 Proteomics Platform, CIC bioGUNE, Basque Research and Technology Alliance (BRTA), ProteoRedISCIII, 48160 Derio, Bizkaia Spain; 4 Centro de Investigación Biomédica en Red de Enfermedades Hepáticas y Digestivas (CIBERehd), Instituto de Salud Carlos III, 28029 Madrid, Spain; 5 Experimental Hepatology Joint Research Unit, IIS Hospital La Fe, Valencia, Spain; 6 Dep. Biochemistry and Molecular Biology, University of Valencia, Valencia, Spain; 7 Chemical Glycobiology Lab, CIC bioGUNE, Basque Research and Technology Alliance (BRTA), 48160 Derio, Bizkaia Spain; 8 Ikerbasque, Basque Foundation for Science, Bilbao, Spain; 9 Department of Organic Chemistry, University of the Basque Country, UPV/EHU, 48940 Leioa, Spain; 10 Centro de Investigación Biomédica En Red de Enfermedades Respiratorias (CIBERES), 28029 Madrid, Spain; 11 Department of Physiology, Center for Research in Molecular Medicine and Chronic Diseases (CIMUS), University of Santiago de Compostela-Instituto de Investigación Sanitaria, CIBER Fisiopatología de a Obesidad y Nutrición (CIBERobn), Galician Agency of Innovation (GAIN), Xunta de Galicia, 15782 Santiago de Compostela, Spain; 12 Univ. Lille, Inserm, CHU Lille, Development and Plasticity of the Neuroendocrine Brain Lab, UMR-S1172 INSERM, DISTALZ, EGID, Lille, France

**Keywords:** Metabolic disorders, Immunology

## Abstract

Severe acute respiratory syndrome coronavirus 2 (SARS-CoV-2) causes a multi-organ damage that includes hepatic dysfunction, which has been observed in over 50% of COVID-19 patients. Liver injury in COVID-19 could be attributed to the cytopathic effects, exacerbated immune responses or treatment-associated drug toxicity. Herein we demonstrate that hepatocytes are susceptible to infection in different models: primary hepatocytes derived from humanized angiotensin-converting enzyme-2 mice (hACE2) and primary human hepatocytes. Pseudotyped viral particles expressing the full-length spike of SARS-CoV-2 and recombinant receptor binding domain (RBD) bind to ACE2 expressed by hepatocytes, promoting metabolic reprogramming towards glycolysis but also impaired mitochondrial activity. Human and hACE2 primary hepatocytes, where steatosis and inflammation were induced by methionine and choline deprivation, are more vulnerable to infection. Inhibition of the renin-angiotensin system increases the susceptibility of primary hepatocytes to infection with pseudotyped viral particles. Metformin, a common therapeutic option for hyperglycemia in type 2 diabetes patients known to partially attenuate fatty liver, reduces the infection of human and hACE2 hepatocytes. In summary, we provide evidence that hepatocytes are amenable to infection with SARS-CoV-2 pseudovirus, and we propose that metformin could be a therapeutic option to attenuate infection by SARS-CoV-2 in patients with fatty liver.

## Abstract (web summary)

SARS-CoV-2 pseudovirus infects human hepatocytes leading to metabolic reprogramming towards glycolysis and impaired mitochondrial activity, and metformin can reduce infection under steatotic conditions.

## Introduction

Severe acute respiratory syndrome coronavirus 2 (SARS-CoV-2) has caused to date, almost 500 million infections worldwide (covid19.who.int). Notably, liver damage is emerging as a coexisting symptom reported in patients with COVID-19^1^. Approximately 50% of COVID-19 patients show elevated transaminase levels, which is a prognostic factor for disease severity^1–3^. The high prevalence of liver damage in patients with COVID-19 may be due to several factors. Antiviral treatments used in these patients, such as arbidol and lopinavir/ritonavir, among others, might partially explain the liver injury described in clinical studies performed in COVID-19 patients^4^. In addition, administration of antibiotics, macrolides or quinolones, steroids, and other drugs, could result in idiosyncratic drug-induced liver injury (IDILI), which presents clinical signs compatible with the ones described for COVID-19: liver necrosis, inflammatory response and steatosis^5^.

In this scenario, it is still unclear if the observed liver damage in COVID-19 patients is a direct consequence of SARS-CoV-2 infection of hepatic cells. Other viruses targeting mainly the upper respiratory tract, such as SARS-CoV and MERS-CoV, have shown tropism to the liver^6,7^. Indeed, the presence of coronavirus particles has been found in liver biopsies from COVID-19 patients presenting abnormal hepatic transaminase levels, hepatic apoptosis and mitochondrial swelling^1,8^. A major limitation of these studies is the limited number of samples, making it difficult to evaluate the direct contribution of SARS-CoV-2 infection of hepatic cells to the observed liver damage.

Angiotensin-converting enzyme 2 (ACE2) is the primary cellular entry receptor for SARS-CoV-2 as a result of the direct interaction with the receptor-binding domain (RBD) of the S1 subunit of the spike protein^9–11^. Transmembrane protease serine 2 (TMPRSS2)^12^ and Neuropilin-1 (NRP1)^13,14^ have also been described as host cell entry mediators implicated in the infection of SARS-CoV-2. We have previously shown that in obese patients with metabolic-associated fatty liver disease (MAFLD) and type 2 diabetes (T2D), hepatic levels of *ACE2* and the cellular *TMPRSS2* transcripts were positively correlated with the NAS score of these patients^15,16^.

About 10% of individuals with COVID-19 suffer from chronic liver disease^17^ and present an increase in morbidity and mortality as a result of SARS-CoV-2 infection^18^. In this context, debatable data addressed the beneficial effect of metformin treatment in mortality and progression of COVID-19 patients^19,20^. Metformin remains a common therapeutic option to manage hyperglycemia in T2D patients^21,22^. In addition, it has been suggested that metformin might reduce both the mortality and severity in T2D patients infected with SARS-CoV-2^23,24^, but the mechanism by which metformin might have a beneficial effect on the prognosis of COVID-19 remains largely unexplored.

To better understand the implications of SARS-CoV-2 infection on the liver, we have performed experiments in human primary hepatocytes and in primary hepatocytes derived from ACE2-humanized mice (hACE2)^25^ with pseudotyped lentiviral particles expressing the full-length spike of SARS-CoV-2 in comparison to control virus^26^. Our results determine that primary hepatocytes are susceptible to SARS-CoV-2 infection. Remarkably, ACE2 levels were significantly increased in hepatocytes isolated from hACE2 mice and cultured under NASH-like conditions, leading to increased infection. Additionally, the inhibition of the renin-angiotensin system increases the infection ratio of both human and mouse hepatocytes. Metabolic flux analyses revealed adaptations and dynamic changes in hACE2 and human hepatocytes after infection, resulting in a rewiring at the mitochondrial level and a shift towards glycolytic metabolism. Furthermore, the proteomic landscape of infected hACE2 hepatocytes evidenced antiviral immunity, exacerbated inflammatory responses, and altered mitochondrial processes, such as mitochondrial translation and iron trafficking. Finally, our results uncovered an unidentified mechanism of metformin action in NASH that could explain the beneficial effects of this drug in the prognosis of SARS-CoV-2-infected patients.

## Results

### The spike of SARS-CoV-2 binds to human hepatocytes and hepatocytes derived from humanized ACE2 (hACE2) mice

**Fig. 1:**
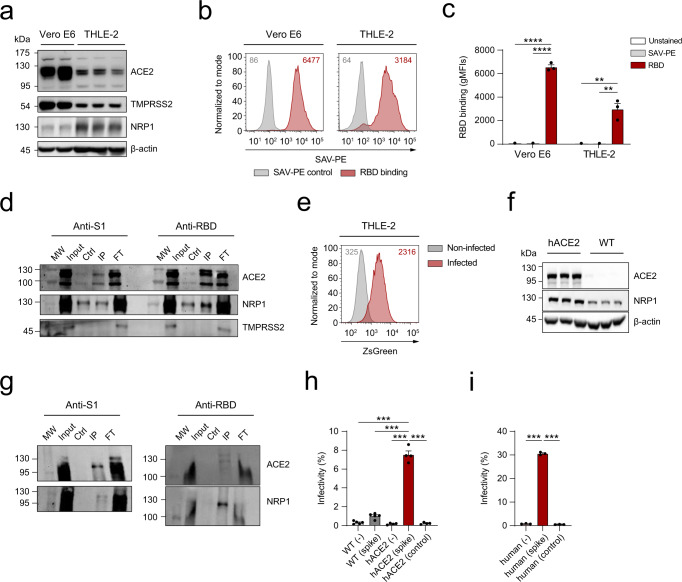
The spike of SARS-CoV-2 binds to human hepatocytes and hepatocytes derived from humanized ACE2 (hACE2) mice. **a** Western blot showing the expression of ACE2, TMPRSS2, and NRP1 proteins on Vero E6 cells (left) or THLE-2 human hepatocytes (right). **b** A representative flow cytometry histogram showing the binding of the RBD to Vero E6 cells (left) or THLE-2 human hepatocytes (right). **c** Binding of the RBD to Vero E6 cells or THLE-2 cells (*n* = 3, unpaired t-test), measured by flow cytometry. **d** Western blot showing the expression of ACE2, NRP1, and TMPRSS2 on different fractions of the immunoprecipitation assay (input, control, immunoprecipitated and flowthrough) with S1 (left) or RBD (right) on THLE-2 human hepatocytes. **e** A representative histogram representing the infection rate of THLE-2 cells measured by flow cytometry (ZsGreen expression) 48 h after addition of the pseudotyped viral particles expressing the full-length spike of SARS-CoV-2. **f** Western blot showing the expression of ACE2, NRP1, and β-actin on hACE2 (left) or WT (right) primary mouse hepatocytes. **g** Western blot showing the expression of ACE2 and NRP1 on the different fractions after the immunoprecipitation assay (input, control, immunoprecipitated and flowthrough) with S1 (left) or RBD (right) on hACE2 primary mouse hepatocytes. **h** Infection rate of hACE2 or WT primary mouse hepatocytes measured by flow cytometry (*n* = 5, one-way ANOVA test). **i** Infection rate of upcyte second-generation human hepatocytes measured by flow cytometry (*n* = 3, one-way ANOVA test). Error bars represent SEM and asterisks represent *p* values (****<0.01, ***<0.001, and ****<0.0001). FT flowthrough in the immunoprecipitation assay, hACE2 humanized angiotensin-converting enzyme 2, IP immunoprecipitated fraction, MW molecular weight marker, SAV-PE Streptavidin-phycoerythrin, WT wildtype.

In order to determine if the spike of SARS-CoV-2 could directly interact with human hepatocytes, the binding capacity of the S1 subunit (S1) or the receptor-binding domain (RBD) were measured by flow cytometry in THLE-2 cells, a human hepatocyte cell line expressing ACE2, TMPRSS2 and NRP1 (Fig. 1a). Figure 1b, c show that RBD interacts with human hepatocytes. Under these conditions, the binding capacity was comparable to that observed on Vero E6 cells^27^, a widely used kidney epithelial cell model in SARS-CoV-2 biology research^28^. In order to confirm that this interaction was mediated by ACE2, we carried out pull-down experiments with S1 or RBD proteins in THLE-2 human hepatocytes (Fig. 1d). This approach confirmed the binding of S1 and RBD to ACE2 in human hepatocytes. We then evaluated the ability of pseudotyped lentiviral particles that express the full-length spike of SARS-CoV-2^26^ to infect human hepatocytes. THLE-2 cells cultured in the presence of pseudotyped viral particles are susceptible to be infected, as detected by flow cytometry (Fig. 1e).

Our findings indicating that human hepatocytes could be a relevant cell target for SARS-CoV-2 prompted us to study its infectivity in relevant primary cell models. For this purpose, we used transgenic mice that express human ACE2 (hACE2) and are therefore SARS-CoV-2 susceptible^25^. We assessed the expression of ACE2 and NRP1, an important factor that mediates the initial steps of SARS-CoV-2 infection, in hACE2- and wild-type (WT)-derived primary hepatocytes by immunoblotting. Both ACE2 and NRP1 protein levels were upregulated in hACE2-derived hepatocytes compared to control hepatocytes (Fig. 1f). Pull-down assays confirmed that S1 and RBD directly bind to ACE2 expressed on primary hepatocytes (Fig. 1g). In order to determine the susceptibility to infection, hACE2 primary hepatocytes were exposed to pseudotyped viral particles expressing the full-length spike of SARS-CoV-2 and pseudotyped viral particles without spike as a negative control. Flow cytometry experiments revealed that hACE2 primary hepatocytes, but not WT mouse hepatocytes, are infected by pseudotyped viral particles expressing the SARS-CoV-2 spike while pseudotyped control viral particles did not show any effect (Fig. 1h). Remarkably, we demonstrate that upcyte second-generation human hepatocytes are also susceptible to infection with pseudotyped viral particles, whereas the control virus had no effect (Fig. 1i). Consistent with these results, we observed that in primary hACE2 hepatocytes, ACE2 levels measured by western blot were decreased in the presence of pseudotyped viral particles, but no modulation was detected upon infection with the control virus (Supplementary Fig. 1a). In human primary hepatocytes, changes in the pattern of ACE2 expression were observed after infection (Supplementary Fig. 1b). NRP1 is another receptor known to enhance viral infection^29^. In this context, we explored NRP1 expression in hACE2 and human primary hepatocytes after infection (Supplementary Fig. 1a,b). Finally, to assess whether infection renders primary mouse and human hepatocytes susceptible to apoptotic processes, we measured caspase-3 activity by fluorimetry in the presence of pseudotyped viral particles. Infection induced an apoptotic response that was dependent on the presence of the spike (Supplementary Fig. 1c).

### Proteomic analyses reveal changes in hACE2 mouse hepatocytes after infection with pseudotyped viral particles expressing the spike of SARS-CoV-2

Significant molecular changes have been reported in cells that have been infected with SARS-CoV-2^30^. In the context of liver infection, we characterized changes at the proteome level that result from the interaction of the full-length spike of SARS-CoV-2 with hACE2 primary hepatocytes.

**Fig. 2:**
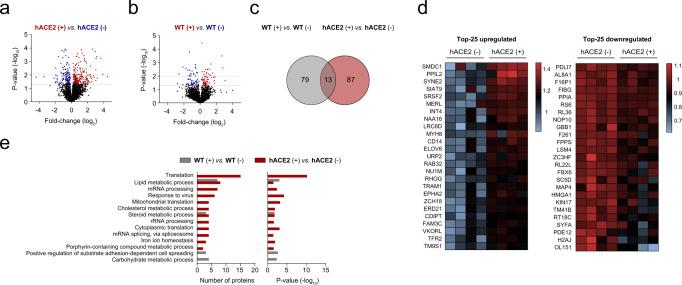
Proteomic analyses reveal changes in hACE2 mouse hepatocytes after infection with pseudotyped viral particles expressing the spike of SARS-CoV-2. **a** Volcano plot showing the 354 differentially expressed peptides between hACE2 mouse hepatocytes in the presence (+) or absence (−) of pseudotyped viral particles after 48 h. **b** Volcano plot showing the 132 differentially expressed peptides between WT mouse hepatocytes in the presence (+) or absence (−) of pseudotyped viral particles. For a detailed list of all peptides, including their fold-change and *p* values refer to the Supplementary Data 1. **c** Venn diagram showing common peptides between hACE2 (+ vs. −) and WT (+ vs. −) mouse hepatocyte comparisons. **d** Heatmap showing the top-25 up- or downregulated peptides between hACE2 mouse hepatocytes in the presence (+) or absence (−) of pseudotyped viral particles. **e** Gene ontology term enriched pathways representing the unique differentially expressed peptides in the WT (+ vs. −) or hACE2 (+ vs. −) mouse hepatocyte comparisons. The number of proteins belonging to the identified dysregulated pathways (left) and their corresponding *p* values (right) are shown.

First, we examined the proteomic changes induced by pseudotyped viral particles expressing the full-length spike of SARS-CoV-2 on primary mouse hepatocytes expressing humanized hACE2 (Fig. 2a) or WT mouse hepatocytes (Fig. 2b). Consistent with the rather absence of infection of WT hepatocytes in comparison with that of the humanized hACE2 model (Fig. 1h), WT hepatocytes presented a lower number of differentially expressed peptides than the humanized hACE2 model (Fig. 2c). In order to focus on changes derived from the binding of the spike of SARS-CoV-2, 13 common peptides that were found significantly dysregulated in an ANOVA + Tukey test (*p* < 0.05) in both WT and hACE2 hepatocytes after exposure to pseudotyped viral particles were discarded in downstream analyses (Fig. 2c). Figure 2d shows the list of the top-25 up- and down-regulated specific peptides in hACE2 hepatocytes after exposure to pseudotyped viral particles. The Database for Annotation, Visualization and Integrated Discovery (DAVID) was used to identify the major pathways altered as a result of the infection. We considered those pathways that were found altered in the hACE2 model but not in the WT model (Fig. 2e). Identified dysregulated pathways included viral-related responses: response to the virus, inflammatory and immune-related signals against infection, receptor-mediated endocytosis and mitochondrial translation. In the context of liver-related pathways, the cholesterol steroids pathway was significantly altered on hACE2 hepatocytes. Moreover, two pathways related to iron homeostasis were significantly upregulated in infected hACE2 hepatocytes compared to control hepatocytes (Fig. 2e). Iron-trafficking proteins such as neutrophil gelatinase-associated lipocalin (NGAL), transferrin endocytosis, and recycling molecules, such as transferrin receptor protein 2 (TFR2) and the transmembrane protease serine 6 (TMPS6), among others, were greatly representative in hACE2 hepatocytes exposed to the pseudotyped viral model (Supplementary Data 1). These results are in line with previous studies describing the role of iron metabolism in COVID-19 patient progression^31–35^. Additionally, KEGG pathways represent the unique peptides in the infected hACE2 hepatocytes compared to control hepatocytes identified ribosome- and COVID-19-related processes (Supplementary Fig. 2a).

### Binding of the spike of SARS-CoV-2 alters mitochondrial activity and glucose homeostasis in mouse and human primary hepatocytes

Iron uptake is essential for the correct functioning of mitochondria; therefore, accumulation of this ion usually leads to oxidative stress^36,37^. In order to clarify whether the infection of hACE2 hepatocytes by pseudotyped viral particles expressing the full-length spike of SARS-CoV-2 affected mitochondrial function, we performed metabolic analyses to evaluate alterations in energy production pathways.

**Fig. 3:**
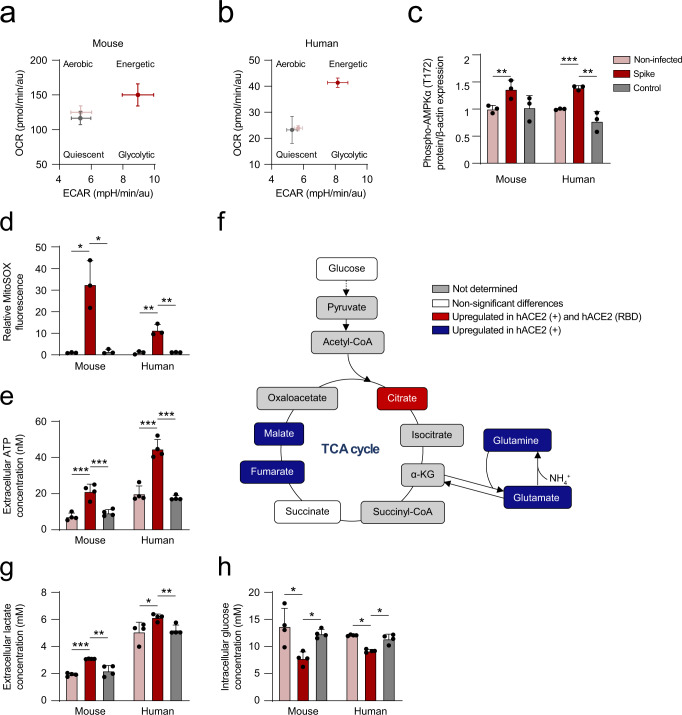
Binding of the spike of SARS-CoV-2 alters mitochondrial activity and glucose homeostasis in hACE2 and human primary hepatocytes. **a**, **b** Oxygen consumption rate (OCR) and extracellular acidification rate (ECAR) after infection with pseudotyped viral particles expressing the spike protein or control of hACE2 hepatocytes (*n* = 6) (**a**) or human primary hepatocytes (*n* = 6) (**b**) measured by extracellular flux analyses. **c** Relative quantification of phospho-AMPKα (T172) levels measured by immunoblotting on primary hACE2 hepatocytes or primary human hepatocytes infected with pseudotyped lentiviral particles (*n* = 3, one-way ANOVA test). **d** Relative MitoSOX fluorescence (*n* = 3, one-way ANOVA test) and **e** extracellular ATP concentration (*n* = 4, one-way ANOVA test) of the indicated groups. **f** Schematic representation showing the up- or down-regulation of metabolites of the TCA cycle after hepatocyte infection with lentiviral particles or in the presence of recombinant RBD (*n* = 3, one-way ANOVA). **g** Extracellular lactate concentration (*n* = 4, one-way ANOVA test) and **h** intracellular glucose concentration (*n* = 4, one-way ANOVA test) of the indicated groups. Asterisks represent *p* values (*<0.05, **<0.01, and ***<0.001).

Figure 3a shows changes in the oxygen consumption rate (OCR) and extracellular acidification rate (ECAR) after infection of hACE2 hepatocytes by pseudotyped viral particles compared to controls, measured by extracellular flux analysis. These results showed a shift towards enhanced glycolysis. Viral infection also enhanced the OCR, suggesting increased mitochondrial activity (Fig. 3a). Importantly, upcyte second-generation human hepatocytes showed a similar metabolic switch characterized by a more energetic state upon infection (Fig. 3b). In this context, the activity of AMP kinase, a major hub for metabolic control in the cell, was examined by immunoblotting in hACE2 and human primary hepatocytes, resulting in an increase in AMPKα phosphorylation in Thr172. No effect was observed after infection with control viral particles (Fig. 3c and Supplementary Fig. 3a). These metabolic changes could result from the direct interaction of the SARS-CoV-2 spike with the ACE2 receptor on the surface of hACE2 and human hepatocytes. In this context, it is known that ACE2 can regulate mitochondrial function and that ACE2-knockout mice show impaired mitochondrial respiration^38–40^. Moreover, exacerbated mitochondrial activity could alter the cell's oxidative state, leading to cell death^41,42^. We also measured the content of mitochondrial reactive oxygen species (ROS) in hACE2 and human primary hepatocytes, showing that infection with pseudotyped viral particles increased ROS production (Fig. 3d and Supplementary Fig. 3b). Consistently, *Tnf* and *Il6* in primary hACE2 hepatocytes (Supplementary Fig. 4a) and *TNF* in case of human primary hepatocytes (Supplementary Fig. 4b) were significantly modulated under pseudovirus S1 infection while no regulation was identified with the control virus. Importantly, iron metabolism was also modulated in response to the lentiviral particles expressing spike. Iron transporters (*TRFC1* and *TRFC2*) were upregulated while *FTL* expression was reduced, suggesting a higher iron utilization by the mice and human primary hepatocytes (Supplementary Fig. 4a, b).

Finally, under these circumstances, extracellular ATP levels were significantly increased in infected primary hepatocytes (Fig. 3e), whereas intracellular ATP levels remained unaltered (Supplementary Fig. 3c).

In order to further understand the rewiring of energy metabolism produced by the binding of the spike of SARS-CoV-2, we performed metabolic flux experiments employing labeled [U-^13^C]-glucose analyzed by high-resolution mass spectrometry on hACE2 hepatocytes. Determination of isotopic enrichment labeled tracer of [U-^13^C]-glucose revealed an increase of the glycolytic flux into the tricarboxylic acid (TCA) cycle in hACE2 hepatocytes after infection with pseudotyped viral particles or binding of recombinant RBD (Fig. 3f). Supplementary Fig. 5a shows that binding of the spike or RBD to hepatocytes increases cellular consumption of labeled glucose, resulting in the activation of downstream energy pathways characterized by accumulation of labeled metabolites that include citrate, fumarate and malate. The observed increase in glutamate levels probably acts to potentiate the activity of the TCA through α-ketoglutarate (α-KG) (Fig. 3f). We also analyzed each labeled carbon independently (Supplementary Fig. 5b), further confirming the findings on rewiring at the mitochondrial level and a shift towards the glycolytic processes shown in Fig. 3a. TCA cycle activity could also be evaluated by measuring the ratio between the labeled citrate on different carbons and the sixth labeled carbon of glucose ([U-^13^C_6_]-glucose). Notably, citrate/glucose ratios in hACE2 hepatocytes in presence of pseudotyped viral particles were significantly increased compared to non-infected cells (Supplementary Fig. 5c), confirming a boosted activity of TCA on hepatocytes upon interaction with the spike of SARS-CoV-2. To confirm these data, glucose and lactate levels were examined under infection with pseudotyped and control viral particles in hACE2 and in human primary hepatocytes. The extracellular lactate levels were upregulated in hepatocytes upon infection compared to controls (Fig. 3g). In addition, a reduction in the levels of intracellular glucose was detected (Fig. 3h), consistent with a more pronounced glycolytic phenotype. Furthermore, gene expression of glycolytic enzymes, lactate, and glutamine metabolism (*G6PDH*, *PKLR*, *PFKL*, *LDHA*, *LDHB*, *GLS1*, *GLS2*, and *GLUL*), were upregulated in primary mouse (hACE2) and human hepatocytes upon infection compared to controls (Supplementary Fig. 4a, b).

### Infection of primary hepatocytes regulates the renin-angiotensin system

ACE2 is a key enzyme of the renin-angiotensin system (RAS) that converts angiotensin ANGII to ANG(1–7). A reduction in the presence of ACE2 in the cell membrane alters the balance of RAS toward an increase in ANGII. Therefore, the activation of the ANG(1–7)/Mas receptor is an important mechanism for counteracting the deleterious effects induced by inappropriate increases in the ANGII/AT1 receptor in several diseases^43^.

**Fig. 4:**
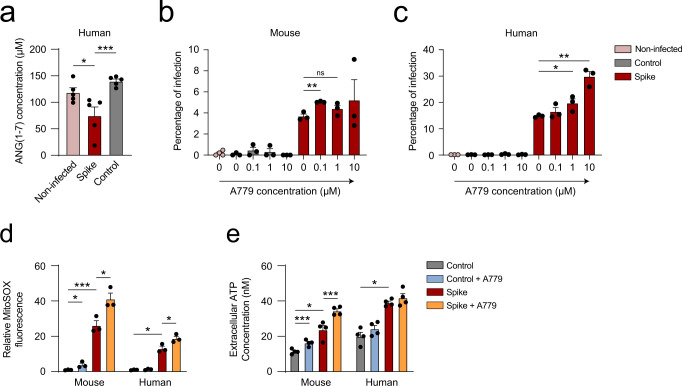
Infection of primary hepatocytes regulates the renin-angiotensin system. **a** Extracellular levels of ANG(1–7) secreted by human primary hepatocytes after infection with pseudotyped viral particles compared to controls, measured by ELISA (*n* = 5, one-way ANOVA). **b**, **c** Percentage of infected hACE2 (**b**) or human (**c**) primary hepatocytes after infection with pseudotyped viral particles or controls in the presence of different doses of A779, measured by flow cytometry (*n* = 3, one-way ANOVA). **d** Relative MitoSOX fluorescence (*n* = 3, one-way ANOVA) and **e** extracellular ATP concentration (*n* = 4, one-way ANOVA test) of the indicated groups. Error bars represent SEM and asterisks represent *p* values (*<0.05, **<0.01, and ***<0.001).

Remarkably, ANG(1–7) levels were significantly reduced in human primary hepatocytes after infection (Fig. 4a), corresponding to ACE2 inhibition after the binding of the spike. Treatment with A779, a pharmacological inhibitor of the ACE2/ANG(1–7)/Mas axis, increased the susceptibility of mouse and human primary hepatocytes to infection (Fig. 4b, c). No apoptotic response was detected at 0.1 and 1 µM concentrations of A779 (Supplementary Fig. 6a, b). These results are consistent with previously observed data showing that an imbalance of ANGII/ANG(1–7) influences susceptibility to SARS-CoV-2 infection^44^. Treatment with A779 increased mitochondrial ROS in mouse and human hepatocytes upon infection (Fig. 4d and Supplementary Fig. 6c). Accordingly, significant changes were also observed in the level of ATP production upon treatment with A779 (Fig. 4e). The blockade of the RAS system in primary hepatocytes by using the Mas inhibitor A779 showed comparable metabolic effects to those observed upon infection with pseudotyped lentiviral particles expressing the spike of SARS-CoV-2 (Fig. 3d, e). During infection, an interaction between the spike and ACE2 occurs, inhibiting RAS signal transduction. Thus, activation of the Mas receptor may counteract the inflammatory response mediated by SARS-CoV-2 infection in primary hepatocytes.

### Dysregulation of hepatocyte mitochondrial activity modulates ACE2 levels and increases susceptibility to infection

Metabolic alterations associated with obesity, fatty liver disease, and T2D have been described as risk factors for COVID-19^45^. In this context, understanding the broad spectrum of factors that shape SARS-CoV-2 pathophysiology in patients with metabolic-associated fatty liver disease (MAFLD) remains a pressing issue. We have recently reported that the liver of patients with MAFLD presents increased levels of *ACE2* and *TMPRSS2*^15^.

**Fig. 5:**
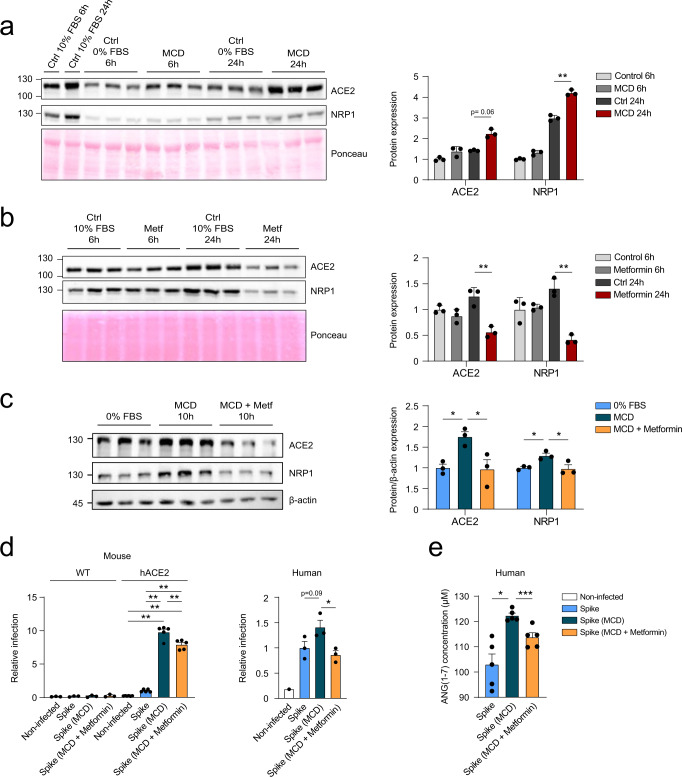
Dysregulation of hepatocyte mitochondrial activity modulates ACE2 levels and increases susceptibility to infection. **a** Western blot showing the modulation of ACE2 and NRP1 protein expression when incubated with methionine-choline deficient (MCD) medium for 6 and 24 h (along with their respective controls. Quantification of ACE2 or NRP1 protein expression by densitometry is also shown (*n* = 3, unpaired *t*-test). **b**, **c** Western blot showing the expression of ACE2 and NRP1 protein expression when treated with 1 mM metformin for 6 or 24 h (*n* = 3, unpaired *t*-test) (**b**) or with MCD or MCD plus 1 mM metformin for 10 h (*n* = 3, one-way ANOVA) (**c**) as well as their respective controls. Relative quantification of ACE2 or NRP1 protein expression (related to β-actin) is also shown. **d** Relative infection with pseudotyped viral particles or controls of WT or hACE2 mouse hepatocytes (left) or upcyte second-generation human hepatocytes (right) upon treatment with MCD or MCD and metformin, measured by flow cytometry (one-way ANOVA). **e** Extracellular levels of ANG(1–7) secreted by human primary hepatocytes after infection with pseudotyped viral particles upon treatment with MCD or MCD and metformin, measured by ELISA (*n* = 5, one-way ANOVA). Error bars represent SEM and asterisks represent *p* values (*<0.05, **<0.01, and ***<0.001).

In order to provide new insights related to the regulation of the viral entry points in the liver, primary hepatocytes isolated from a humanized ACE2 mouse model were exposed to steatotic insults. ACE2 levels were significantly elevated in a time-dependent manner in hACE2 hepatocytes cultured in a medium deficient in methionine and choline (MCD) that triggers the accumulation of lipids and the production of ROS, generating mitochondrial dysfunction^46^. Additionally, NRP1, which is known to bind furin-cleaved substrates and potentiate SARS-CoV-2 infectivity, was also induced in steatotic hepatocytes (Fig. 5a). Indeed, NRP1 has a critical role in liver fibrosis and cirrhosis pathogenesis^47,48^.

Metformin is an antidiabetic drug widely used in patients with MAFLD^49^. Metformin acts by inhibiting the activity of Complex I of the mitochondrial respiratory chain, directly modulating ATP production in the cell^50,51^. Recently, metformin has been proposed as a potential therapy for COVID-19 patients^23,24^, although its role in clinical disease progression remains controversial. Therefore, we have evaluated if metformin has any impact on the success of the viral entry in hACE2 hepatocytes. Treatment with metformin significantly reduced the levels of ACE2 and NRP1 proteins in hACE2 hepatocytes in a time-dependent manner (Fig. 5b). Notably, metformin was able to diminish the levels of ACE2 and NRP1 in stimulated steatotic hepatocytes to a similar content of that of healthy hACE2 liver cells (Fig. 5c). A similar result was observed at the transcriptional level of *Ace2* and *Nrp1* (Supplementary Fig. 7a). Importantly, the expression of *Tmprss2* was induced in steatotic conditions and reduced upon treatment with metformin (Supplementary Fig. 7a). These results were also examined in human primary hepatocytes under steatotic conditions and in the presence or absence of metformin (Supplementary Fig. 7b). Induction of *ACE2, NRP1 and TMPRSS2* expression under steatotic conditions were identified, as described previously^15^ (Supplementary Fig. 7b). In these conditions, metformin was able to reduce their expression (Supplementary Fig. 7b).

Steatotic hACE2 hepatocytes exposed to pseudotyped viral particles expressing the spike of SARS-CoV-2 showed higher susceptibility to infection compared to healthy cells (Fig. 5d). Metformin is able to significantly reduce the increased infection rate observed in metabolically compromised hepatocytes. Human primary hepatocytes showed a similar response in cells treated with MCD in the presence or absence of metformin (Fig. 5d). Finally, we studied the effect of metformin on the RAS system upon infection with pseudotyped lentiviral particles expressing the spike of SARS-CoV-2 (Fig. 5e). Levels of ANG(1–7) measured by ELISA were upregulated under steatotic conditions (Fig. 5e). Activation of the counterregulatory ACE2/ANG(1–7)/Mas axis has been shown to prevent liver injury^52^. Therefore, metformin significantly reduced ANG(1–7), likely due to the reduction in oxidative stress and thus reduced liver injury (Fig. 5e and Supplementary Fig. 7a, b). According to this, *TNF* and *IL-6* gene expression were upregulated when metformin was present (Supplementary Fig. 7a, b).

## Discussion

The impact of SARS-CoV-2 on the liver has been extensively debated. The high incidence of COVID-19 patients with elevated transaminases may be due to a compromised detoxifying capacity of the liver in these poly-treated patients, the response to a proinflammatory environment, or the possible tropism of SARS-CoV-2 to the liver. On the other hand, liver disease is a risk factor for severe COVID-19 pathology as described in MAFLD patients^53^. Given the controversial information on SARS-CoV-2 tropism to the liver, we have demonstrated that pseudotyped viral particles expressing the full-length spike of SARS‐CoV‐2 were able to infect human primary hepatocytes as well as hepatocytes isolated from a humanized hACE2 mouse model. Mass spectrometry analysis revealed that in primary hepatocytes infected with pseudotyped lentiviral particles expressing the spike of SARS‐CoV‐2, the main molecular processes dysregulated were mitochondria-dependent. Specifically, fluxomics analyses revealed an increase in the TCA activity in hACE2 and human primary hepatocytes as a result of infection with pseudotyped viral particles or binding of recombinant RBD, indicating a shift to a glycolytic phenotype induced by ACE2 after binding of the spike. In the present study, we have also dissected hepatic expression patterns of SARS-CoV-2 viral entry points under steatotic conditions, demonstrating a higher susceptibility to infection of steatotic hepatocytes. Finally, we have reported new insights into the beneficial effects of metformin treatment in patients with MAFLD as a result of a significant reduction of hepatic ACE2 content and reduced predisposition to infection.

Several reports provided the first data supporting the notion that hepatic cells are permissive for SARS‐CoV-2 infection and viral replication^54–56^, but the usage of immortalized hepatoma cell models instead of primary hepatocytes limited the depth of their findings. More recently, several studies have provided further evidence by using organoids derived from human hepatocytic stem cells demonstrating that the liver could be a potential target of SARS‐CoV-2^57–59^. Although other hepatic cell types like cholangiocytes showed higher expression of ACE2 than hepatocytes, no direct evidence of infection in these cells have been reported in the liver of patients with COVID-19^59^ in spite of the fact that viral particles compatible with SARS-CoV-2 were identified by electron microscopy techniques in the hepatocytes from COVID-19 autopsies^1^. These data encourage a rigorous study of the permissiveness of the liver to SARS-CoV-2 infection and its potential cytopathic effect that could explain the deleterious effects identified in COVID-19 patients. The models presented in this study include a non-transformed human hepatocyte cell line, primary hepatocytes obtained from humanized hACE2 transgenic mice and upcyte second-generation human hepatocytes. These approaches demonstrate that the spike of SARS-CoV-2 binds to ACE2 expressed in primary hepatocytes and identify the hepatocyte as a susceptible cell target for SARS-CoV-2 infection. This information may be relevant for the clinical treatment of COVID-19 patients, considering that liver functionality may be compromised by infectivity.

Furthermore, our approach by mass spectrometry analysis revealed a molecular mechanism in hACE2 hepatocytes that compromised mitochondrial activity as a result of infection. Previous data have shown that the depletion of the Angiotensin-(1–7) receptor *Mas* in hepatocytes aggravates mitochondrial dysfunction, increased mitochondrial ROS, induced fatty acid synthesis, and impaired cholesterol synthesis/efflux^39^. Hence, lipid alteration and insulin resistance in the hepatocytes, concomitant with an apoptotic process was observed in the hepatocytes *Mas*^−/−^. These data are consistent with our proteomics results where a possible ACE2 inhibition mediated mitochondrial dysfunction has been detected. Indeed, iron metabolism appeared significantly overrepresented in infected hACE2 hepatocytes. Increased iron uptake upon viral infection would increase mitochondrial ROS^37^. Accordingly, Saleh et al. described that severity in COVID-19 patients was associated with hyperferritinemia^60^. Our data sustain the idea that this misbalance in iron metabolism and mitochondrial ROS production could partly result from the alteration in the cellular oxidative homeostasis promoted by an exacerbated mitochondrial activity in infected mouse and human primary hepatocytes, triggering an apoptotic response.

A metabolic reprogramming similar to the Warburg effect in cancer cells has been previously identified in the host cells under SARS-CoV-2 infection^61^. Fluxomics analyses revealed that infected hACE2 hepatocytes present a glycolytic phenotype characterized by higher production of lactic acid and a more active TCA. Experiments performed in human primary hepatocytes further evidenced a modulation of intracellular glucose and extracellular lactate upon infection. These data are consistent with previous work showing that viral infection promotes glycolysis^62^. Remarkably, in our fluxomics results performed with the primary hepatocytes, infection resulted in an increase of fumarate and malate. This altered glycolytic metabolism could support the replication of the virus in the liver and may also contribute to evading cytotoxic immune responses by acidifying the extracellular compartment.

Infection of hACE2 hepatocytes resulted in disturbances in glucose and glutamine metabolism. In these circumstances, enhancement of glutaminolysis metabolism with upregulation of glutamate levels and triggering of TCA was also detected. This metabolic pathway has been identified as a key player in infected host cells, as previously reported for other viruses, and may trigger replication of SARS-CoV-2^63^. Our group has previously described the alteration of this anaplerotic pathway with upregulation of the enzyme glutaminase 1, which is responsible for glutamine catabolism in the liver of patients with NAFLD^64^. Therefore, there might be a glutamine-dependent link between liver disease and SARS-CoV-2 infection.

The described metabolic switch was mediated in part by inhibition of the RAS system through the interaction between the spike and ACE2. Indeed, inhibition of the Mas receptor with A779 amplified the observed phenotype. MAFLD patients showed elevated levels of *ACE2* and *TMPRSS2* in the liver^15,16^. Interestingly, high levels of Angiotensin-(1–7), the product of ACE2, were able to revert MAFLD through the activity of its receptor *Mas*, modulating hepatic mitochondrial function. Our data suggest a compensatory mechanism that may help to prevent impaired mitochondrial activity and liver damage. Although this response may initially be beneficial for the hepatocyte, our results identified a higher susceptibility to infection. These data support the predisposition of MAFLD patients to a more severe COVID-19 prognosis.

Metformin is a widely used pharmacological choice for the treatment of hyperglycemia in MAFLD patients. Beyond its effect on glucose metabolism, metformin improves mitochondrial respiration, reducing Complex I activity through the activation of 5’ AMP-activated protein kinase (AMPK)^65^. Additionally, metformin has been associated with the modulation of post-translational modifications, including ubiquitination of several target proteins^66,67^. Thus, the effects of metformin were evaluated in human and hACE2 primary hepatocytes under steatotic conditions. This approach revealed downregulation of ACE2 levels in hepatocytes treated with metformin, resulting in resistance to infection. Importantly, ANG(1–7) levels were reduced under metformin treatment, probably as a reduction of oxidative stress and reduced ACE2 levels. These data suggest that metformin treatment could be beneficial for COVID-19 patients previously diagnosed with MAFLD and T2D.

In summary, our findings obtained from different models demonstrate that hepatocytes are susceptible to infection, and upon entrance of the virus, they experience a metabolic reprogramming towards glycolysis but also mitochondrial dysfunction. Steatotic hepatocytes are more vulnerable to infection, and under this context, metformin might prevent liver dysfunction caused by SARS-CoV-2.

## Methods

### Cell lines and human upcyte hepatocytes

Human liver epithelial THLE-2 cells (ATCC Cat#: CRL-2706) were cultured under standard conditions (37 °C and 5% CO_2_) in Bronchial Epithelial Cell Growth medium (Lonza Cat#: CC3170) supplemented with 10% FBS (Gibco Cat#: 10270106), all items included in the BulletKit (Lonza Cat#: CC4175), except for Gentamycin/Amphotericin (GA) and Epinephrine, 1% PSA (Thermo Fisher Cat#: 15240062), 1% glutamine (Thermo Fisher Cat#: 25030024), 5 ng/mL EGF (Thermo Fisher Cat#: PHG0315) and 70 ng/mL Phosphorylethanolamine (Sigma Aldrich Cat#: P0503). Vero E6 cells (ATCC Cat#: CRL-1586) were kindly provided by Nicola G.A. Abrescia (CIC bioGUNE) and cultured in MEM (Gibco Cat#: 31095-029) supplemented with 10% FBS (Thermo Fisher Cat#: 10270106) and 1% Penicillin-Streptomycin (P/S) (Thermo Fisher Cat#: 15140122). HEK293T cells (Takara Bio Inc. Cat#: 632180) were cultured in DMEM (Gibco Cat#: 41966-029) supplemented with 10% FBS and 1% P/S. Second-generation human upcyte hepatocytes, culture medium, high-performance medium, and thawing medium were all obtained from Upcyte Technologies (Heidelberg, Germany) and cultured as previously described^68^.

### Mouse models

All mouse experiments were carried out following the ethical guidelines established by the Biosafety and Welfare Committee at CIC bioGUNE (P-CBG-CBBA-0518). Humanized ACE2 (hACE2) and wild-type (WT) mice (male, 18–20 weeks) were kindly provided by the Development & Plasticity of the Neuroendocrine Brain laboratory (Institut National de la Santé et de la Recherché Médicale, INSERM). They were maintained on a 12/12 h light/dark cycle at a temperature of 21 ± 1 °C, the humidity of 45 ± 10%, and ad libitum access to water and a standard chow diet (Teklad Global 14% Protein Rodent Maintenance diet; Envigo RMS Spain Cat#: 2014C). All animal experiments were performed according to the ARRIVE guidelines and carried out in accordance with the National Institutes of Health guide for the care and use of Laboratory animals (NIH Publications N0.8023, revised 1978) and the guidelines of the European Research Council for animal care and use.

### Isolation of primary hepatocytes

Isolation of primary hepatocytes was performed as previously described^64^. Briefly, after anesthesia of hACE2 and WT mice with isoflurane (1.5% isoflurane in O_2_) and insertion of a catheter into the vena cava, the liver was perfused with buffer A (1X PBS, 5 mM EGTA) (37 °C, oxygenated) while portal vein was cut. Buffer B (1X PBS, 1 mM CaCl2, collagenase type I (Worthington)) (37 °C, oxygenated) was subsequently used to perfuse and disaggregate the liver. Then, the disaggregated liver was placed in a Petri plate containing 10%-FBS MEM medium supplemented with PSG and cells were disassembled with the help of forceps and filtered through sterile gauze. Perfused livers were first centrifuged at 400 RPM for 4 min at 4 °C and the pellet was resuspended in 10%-FBS Minimum Essential Medium (MEM) (Gibco Cat#: 31095-029) containing penicillin (100 U/mL), streptomycin (100 U/mL) and glutamine (2 mM) (PSG) (Thermo Fisher Cat#: 10378-016). Subsequently, cells were washed in MEM twice (500 RPM for 5 min at 4 °C) and plated on a collagen-coated plate. Primary hepatocytes were cultured under standard conditions (37 °C and 5% CO_2_).

### Generation of pseudotyped viral particles

In order to generate pseudotyped viral particles expressing the spike protein of SARS-CoV-2, HEK293T cells were transfected using a third-generation five-plasmid system kindly provided by Drs. Jean-Philippe Julien (University of Toronto) and Jesse D. Bloom (Fred Hutchinson Cancer Research Center), as previously described^26^. Briefly, plasmids encoding for HDM-Hgpm2 (NR-52517), pRC-CMV-Rev1b (NR-52519), HDM-tat1b (NR-52518), the SARS-CoV-2 spike protein (NR-52514), and the lentiviral backbone that express ZsGreen (NR-52520) were administered to HEK293T cells (50–70% of confluence) using JetPEI kit (Polyplus-transfection #101-10 N). Pseudotyped lentiviral particles were collected from supernatants 48 h after transfection and filtered using a 0.45 µm filter (VWR Cat#: 514-0063). After concentrating the viral particles using Lenti-X Concentrator (Takara Bio Inc. Cat#: 631231), they were stored in PBS at −80 °C until use. Control pseudotyped viral particles were generated using HEK293T cells. About 5 × 10^6^ cells were seeded in T175 flasks in DMEM media supplemented with 10%c FBS, glutamine and NEAA. For transfection, a mixture of plasmids encoding for luciferase IRES ZsGreen, HDM-Hgpm2, pRC-CMV-Rev1b, and HDM-tat1b were diluted in 2.5 mL of DMEM and then mixed with additional 2.5 mL of DMEM containing lipofectamine. After 24 h, the media was replaced with complete media supplemented with 5 mM of sodium butyrate. After 60 h, viral particles were collected and stored at −80 °C.

### Titration of pseudotyped lentiviral particles and infection of hepatocytes

Viral titration was performed in THLE-2 and upcyte second-generation human hepatocytes cells as described in ref. ^26^. Cells were incubated with pseudotyped lentiviral particles for 48 h. The infected cells expressed ZsGreen, allowing their detection by flow cytometry.

### THLE-2 and primary hepatocyte treatments

Rates of infection on THLE-2 and primary hACE2 or WT hepatocytes were calculated after incubation with pseudotyped viral particles (MOI: 0.8). Cells were incubated for 48 h to allow expression of ZsGreen, which allows detection of infected cells by flow cytometry. Steatotic conditions were induced in primary hepatocytes as follows: incubation overnight on 0%-FBS MEM medium containing PSG followed by incubation in methionine-choline deficient (MCD) medium (Gibco Cat#: ME120128L1) supplemented with PSG. Metformin (Sigma Aldrich Cat#: PHR1084) was administered at 1 mM (final concentration) to primary hepatocytes after overnight incubation with 10%-FBS MEM containing PSG. After 6 or 24 h, plates were washed thrice with PBS, frozen (−80 °C), and processed for western blot analysis. Hepatocytes were cultured overnight with 0%- FBS MEM (containing PSG) prior to incubation in an MCD medium containing metformin (1 mM) for 10 h. Hereafter, plates were washed thrice with PBS, frozen (−80 °C), and processed for western blot analysis or incubated with pseudotyped viral particles for 48 h. For pharmacological inhibition of the ACE2/ANG(1–7)/Mas axis, cells were treated with A779 (Tocris Bioscience Cat#: 5937) for 1 h, followed by lentiviral infection.

### Pull-down assay

Cells were resuspended in RIPA lysis buffer (ddH2O, 100 mM NaCl, 1.6 mM Na2HPO4, 8.4 mM NaH2PO4, 0.5% (w/v) sodium deoxycholate, 0.1% (w/v) SDS and 0.005% (w/v) sodium azide, 1 μM sodium orthovanadate, 50 mM sodium fluoride, and Triton X-100 0.1%), sonicated and centrifuged at 14,000 RPM for 30 min at 4 °C. The pellet was discarded. Of total supernatant, 50 μL were kept for use as the control in the western blot assay. After quantification, 1180 μg of protein were incubated with 2 μg of biotinylated S1 (Acrobiosystems Cat#: S1N-C82E8) or RBD (Acrobiosystems Cat#: SPD-C82E9) recombinant proteins for 2 h at 4 °C with constant inversion. Then, 50 µL of prewashed High Capacity Neutravidin beads (Thermo Fisher Cat#: 11805845) were added to the mix and incubated for an additional 30 min at 4 °C with constant inversion. After centrifugation of beads, supernatants were kept as flowthrough inputs for the western blot. Proteins bound to the beads were washed with RIPA buffer for 5 min at RT with constant inversion and eluted in 17 µL of 5X Laemmli buffer. Outputs from the pull-down assay were boiled for 10 min at 95 °C prior to the western blot assay.

### Western blot

Cell lysates were collected with RIPA lysis buffer and quantified using a micro-BCA kit (Thermo Fisher Cat#: 10249133). Samples were boiled for 10 min at 95 °C in 5x Laemmli buffer, separated in an SDS-PAGE gel, and transferred to a polyvinylidene difluoride membrane. Membranes were incubated with 5% non-fat milk in tris-buffered saline buffer (10 mM Tris, pH 8.0, 150 mM NaCl) with 0.1% Tween-20 detergent (TBST) for 1 h and incubated with anti-ACE2 (Bioss Inc Cat #: BS-1004R), anti-NRP1 (Bio-Techne Corporation Cat#: NBP2-67539), anti-TMPRSS2 (Bio-Techne Corporation Cat#: NBP3-00492), anti-Phospho-AMPKα (Thr172) (Cell Signaling Cat#: 2531), and anti-β-actin (Sigma Aldrich Cat#: A5441) primary antibodies at 4 °C for 16 h (dilution 1:1000 or 1:500 for the pull-down assay). Membranes were washed thrice for 10 min and incubated with a secondary anti-rabbit-HRP antibody (Cell signaling Cat#:7074) for 1 h (1:5000 dilution). Blots were washed again with TBST and developed with ECL substrate (Bio-Rad Cat#: 1705061) on an iBright system (Invitrogen). Band densitometry was performed using ImageJ.

### Assessment of binding of recombinant RBD to the cell surface

The binding of recombinant RBD (Acrobiosystems Cat#: SPD-C82E9) to THLE-2 and Vero E6 cells, as well as to primary hepatocytes, was measured as described in ref. ^27^. Briefly, cells were harvested from culture plates using cell dissociation buffer (Thermo Fisher Cat#: 13151-014), counted and 100,000 cells were distributed in 96-well polystyrene conical bottom plates (Thermo Fisher Cat#: 249570). After washing cells with blocking buffer (PBS containing 0.5% BSA; Sigma Aldrich Cat#: A9647), they were incubated with biotinylated RBD (20 μg/mL) for 40 min on ice. After incubation, cells were washed again with blocking buffer and incubated for an additional 15 min with streptavidin-PE (1:200, Thermo Fisher Cat#: 12-4317-87) on ice in a total volume of 100 μL of blocking buffer. Finally, cells were washed twice and resuspended in a blocking buffer containing DAPI (Invitrogen Cat#: D1306) to discriminate alive cells. All centrifugation steps were performed at 300×*g* for 5 min at 4 °C. Cells were acquired on a FACSymphony cytometer (BD Biosciences) and results were analyzed using FlowJo version 10 (BD Biosciences).

### Proteomic analysis by LC-MS/MS

Cells were treated with cell lysis buffer (7 M urea, 2 M thiourea, 4% CHAPS), vortexed, and spun down to remove debris. Extracted protein was digested following the SP3 protocol described by ref. ^69^, with minor modifications. Trypsin was added to a trypsin:protein ratio of 1:10, and the mixture was incubated for 2 h at 37 °C. The resulting peptides were dried out and resuspended in 0.1% formic acid. Samples were analyzed in a novel hybrid trapped ion mobility spectrometry-quadrupole time-of-flight mass spectrometer (timsTOF Pro with PASEF, Bruker Daltonics) coupled online to an EVOSEP ONE (EVOSEP). This mass spectrometer takes advantage of a novel scan mode termed parallel accumulation-serial fragmentation (PASEF), which multiplies the sequencing speed without any loss in sensitivity^70^. Samples (200 ng) were directly loaded in a 15 cm analytical column (EVOSEP) and resolved at 300 nl/min with a 44 min gradient. Protein identification and quantification was carried out using PEAKS software using default settings. Searches were carried out against a database consisting of mice protein entries (Uniprot/Swissprot), with precursor and fragment tolerances of 20 ppm and 0.05 Da. Only proteins identified with at least two peptides at FDR <1% were considered for further analysis. Data was loaded onto Perseus platform^71^ and further processed (log2 transformation, imputation). Statistical analyses between groups were performed with a student’s t-test. Volcano plot and heatmaps showing the 25-top upregulated and downregulated proteins were represented using GraphPad PRISM v8 (GraphPad, San Diego, CA, USA). Enriched Gene Ontology/KEGG pathways were inferred using DAVID (https://david.ncifcrf.gov/home.jsp).

### OCR and ECAR calculation

Oxygen consumption rate (OCR) and extracellular acidification rate (ECAR) was determined as previously described^72^ using a Seahorse XFe24 Analyzer (Seahorse Biosciences). For that, 20,000 primary hepatocytes were plated and incubated with pseudotyped viral particles on an XF24 cell culture microplate (Seahorse Bioscience). Real-time respiration assays were performed after 48 h using DMEM medium (Thermo Fisher Cat#: 12800017) supplemented with glutamine (1 mM), glucose (10 mM), and sodium pyruvate (2 mM) and lacking bicarbonate. After analysis, cell density was measured by crystal violet for data normalization.

### Extracellular l-lactate concentration

l-lactate concentrations were determined in infected or control primary hepatocytes using a commercial kit (Trinity Biotech Cat#: 735-10) following the manufacturer’s recommendations.

### Caspase-3 activity assay

Cells were washed with PBS twice and lysed in 30 µL of caspase-3 reaction buffer (250 mM PIPES pH 7.4, 100 mM EDTA, 2.5% CHAPS, and 125 mM DTT). Total protein was extracted, and protein concentration was determined by the Bradford assay. Forty micrograms of total protein were added to a mix containing 25 μM Ac-DEVD-AFC caspase-3 fluorogenic substrate (ALX-260-032, Enzo Life Sciences) in reaction buffer to a final volume of 500 μL. Each sample was measured in duplicate by adding 200 μL of the reaction mixture to each well of a 96-well black flat bottom assay plate (Corning Cat#: 3915). The reaction plate was incubated at 37 °C with gentle shaking for 4 h and fluorescence (λ_ex_ = 390 nm and λ_em_ = 510 nm) was measured every hour in a SpectraMax M2/M2e microplate reader (Molecular Devices). Caspase-3 activity was determined by calculating the increase in fluorescence from 0 to 4 h after background correction and normalized against the total protein.

### Determination of mitochondrial reactive oxygen species (ROS)

Mitochondrial ROS production in primary hepatocytes was assessed using MitoSOX Red mitochondrial superoxide indicator (Invitrogen Cat #: M36008). Cells were labeled with 2 mM MitoSOX for 10 min at 37 °C in a CO_2_ incubator. After that, cells were washed thrice with PBS. Five to ten random images per sample were taken using an upright fluorescent microscope (Axioimager D1). The percentage of stained areas were calculated using FIJI (ImageJ) and normalized by cell number.

### Extracellular and intracellular ATP concentration

Extracellular and intracellular ATP concentrations were measured using the ATPlite luminescence assay system (PerkinElmer Cat#: 6016943), following the manufacturer’s recommendations and normalizing by total protein content.

### RNA extraction and quantification

RNA extraction and quantification by quantitative PCR (Q-PCR) were performed as previously described^15^. Q-PCR reactions were conducted in triplicate using gene-specific primers (Supplementary Data 2). RNA expression levels were normalized to *Arp* for each sample.

### Metabolic flux analyses

Primary mouse hepatocytes (500,000 cells) were incubated with pseudotyped viral particles expressing the full-length spike of SARS-CoV-2 (MOI: 0.8) or recombinant RBD (20 ug/mL) for 48 h. After that, labeled ^13^C_6_-glucose was added at a 10 mM final concentration for 6 h. For metabolite extraction, 500 µL of cold methanol and water (50/50% v/v) was added to the wells of the culture plates. Plates were left on dry ice for 15 min. Subsequently, 400 µL of the homogenate plus 400 µL of chloroform was transferred to a new aliquot and shaken at 1400 RPM for 1 h at 4 °C. Aliquots were centrifuged for 30 min at 13,000 RPM at 4 °C to separate the organic phase from the aqueous phase. A total of 250 µL of the aqueous phase was transferred to a fresh aliquot and placed at −80 °C for 20 min. Chilled supernatants were evaporated with SpeedVac vacuum concentrators (Thermo Fisher) for 2 h. The resulting pellets were resuspended in 150 µL of water/acetonitrile (40/60% v/v). Samples were measured with a UPLC system (Acquity, Waters Inc., Manchester, UK) coupled with a time-of-flight mass spectrometer (ToF MS, SYNAPT G2, Waters Inc.). A 2.1 × 100 mm, 1.7 µm BEH amide column (Waters Inc.), thermostated at 40 °C, was used to separate the analytes before entering the MS. Mobile phase solvent A (aqueous phase) consisted of 99.5% water and 0.5% FA and solvent B (organic phase) consisted of 4.5% water, 95% MeCN, and 0.5% FA. In order to obtain a good separation of the analytes, the following gradient was used: from 10% A to 99.9% A in 2.6 min in curved gradient (#9, as defined by Waters), constant at 99.9% A for 1.6 min, back to 10% A in 0.3 min. The flow rate was 0.250 mL/min, and the injection volume was 4 µL. After every 12 injections, a QC sample was injected. Samples were injected in duplicate ad random. The MS was operated in negative electrospray ionization full scan mode. The cone voltage was 25 V and capillary voltage was 250 V. Source temperature was set to 120 °C and capillary temperature to 450 °C. The flow of the cone and desolvation gas (both nitrogen) were set to 5 and 600 L/h, respectively. A 2 ng/mL leucine-enkephalin solution in water/acetonitrile/formic acid (49.9/50/0.1 %v/v/v) was infused at 10 µL/min and used for a lock mass which was measured each 36 for 0.5 s. Spectral peaks were automatically corrected for deviations in the lock mass. Extracted ion traces for relevant analytes were obtained in a 20 mDa window in their expected m/z-channels. These traces were subsequently smoothed and peak areas integrated with TargetLynx software. Signals of labeled analytes were corrected for naturally occurring isotopes. The isotope corrected areas were adjusted by median fold-change (MFC) adjustment. This is a robust adjustment factor for global variations in signal due to e.g., differences in tissue amounts, signal drift, or evaporation. The MFC is based on the total amount of detected mass spectrometric features (unique retention time/mass pairs). The calculations and performance of the MFC adjustment factors are described in the following publications^73,74^. Finally, means between duplicates of the adjusted areas were reported.

### Assessment of extracellular angiotensin 1–7 (Ang1–7) levels

Assessment of extracellular angiotensin 1–7 (Ang1–7) levels was performed by ELISA (Cloud-Clone Corp. Cat#: CES085Mi), following the manufacturer’s recommendations.

### Statistics

Statistical analyses were performed using GraphPad PRISM v8 (GraphPad, San Diego, CA, USA). Bar plots show the mean and the standard error of the mean (SEM). Student’s *t*-test or one-way ANOVA test were applied as appropriate (indicated on each figure legend).

## Supplementary information

Peer Review File

Supplementary Information

Description of Additional Supplementary Files

Supplementary Data 1

Supplementary Data 2

Supplementary Data 3

## Data Availability

The mass spectrometry proteomics data have been deposited to the ProteomeXchange Consortium via the PRIDE partner repository with the dataset identifier PXD035263. Uncropped and unedited blot images are provided in Supplementary Fig. 8. All source data underlying the graphs and charts presented in the figures are presented in Supplementary Data 3.

## References

[1] Wang Y (2020). SARS-CoV-2 infection of the liver directly contributes to hepatic impairment in patients with COVID-19. J. Hepatol..

[2] Zhang C, Shi L, Wang FS (2020). Liver injury in COVID-19: management and challenges. Lancet Gastroenterol. Hepatol..

[3] Wagner J (2021). Elevated transaminases and hypoalbuminemia in Covid-19 are prognostic factors for disease severity. Sci. Rep..

[4] Yang X (2020). Clinical course and outcomes of critically ill patients with SARS-CoV-2 pneumonia in Wuhan, China: a single-centered, retrospective, observational study. Lancet Respir. Med..

[5] Fontana RJ (2014). Pathogenesis of idiosyncratic drug-induced liver injury and clinical perspectives. Gastroenterology.

[6] Xu L, Liu J, Lu M, Yang D, Zheng X (2020). Liver injury during highly pathogenic human coronavirus infections. Liver Int..

[7] Kukla, M. et al. COVID-19, MERS and SARS with concomitant liver injury-systematic review of the existing literature. *J. Clin. Med.*10.3390/jcm9051420 (2020). 10.3390/jcm9051420 PMC7290752 32403255

[8] Lagana SM (2020). Hepatic pathology in patients dying of COVID-19: a series of 40 cases including clinical, histologic, and virologic data. Mod. Pathol..

[9] Yan R (2020). Structural basis for the recognition of SARS-CoV-2 by full-length human ACE2. Science.

[10] Walls AC (2020). Structure, function, and antigenicity of the SARS-CoV-2 spike glycoprotein. Cell.

[11] Clausen TM (2020). SARS-CoV-2 infection depends on cellular heparan sulfate and ACE2. Cell.

[12] Hoffmann M (2020). SARS-CoV-2 cell entry depends on ACE2 and TMPRSS2 and is blocked by a clinically proven protease inhibitor. Cell.

[13] Cantuti-Castelvetri L (2020). Neuropilin-1 facilitates SARS-CoV-2 cell entry and infectivity. Science.

[14] Daly JL (2020). Neuropilin-1 is a host factor for SARS-CoV-2 infection. Science.

[15] Fondevila MF (2021). Obese patients with NASH have increased hepatic expression of SARS-CoV-2 critical entry points. J. Hepatol..

[16] Meijnikman AS, Bruin S, Groen AK, Nieuwdorp M, Herrema H (2021). Increased expression of key SARS-CoV-2 entry points in multiple tissues in individuals with NAFLD. J. Hepatol..

[17] Jothimani D, Venugopal R, Abedin MF, Kaliamoorthy I, Rela M (2020). COVID-19 and the liver. J. Hepatol..

[18] Marjot T (2021). COVID-19 and liver disease: mechanistic and clinical perspectives. Nat. Rev. Gastroenterol. Hepatol..

[19] Hariyanto TI, Kurniawan A (2020). Metformin use is associated with reduced mortality rate from coronavirus disease 2019 (COVID-19) infection. Obes. Med..

[20] Lukito AA (2020). The effect of metformin consumption on mortality in hospitalized COVID-19 patients: a systematic review and meta-analysis. Diabetes Metab. Syndr..

[21] Davies MJ (2018). Management of hyperglycaemia in type 2 diabetes, 2018. A consensus report by the American Diabetes Association (ADA) and the European Association for the Study of Diabetes (EASD). Diabetologia.

[22] Sanchez-Rangel E, Inzucchi SE (2017). Metformin: clinical use in type 2 diabetes. Diabetologia.

[23] Yang W, Sun X, Zhang J, Zhang K (2021). The effect of metformin on mortality and severity in COVID-19 patients with diabetes mellitus. Diabetes Res. Clin. Pr..

[24] Varghese E, Samuel SM, Liskova A, Kubatka P, Busselberg D (2021). Diabetes and coronavirus (SARS-CoV-2): molecular mechanism of metformin intervention and the scientific basis of drug repurposing. PLoS Pathog..

[25] Jia H, Yue X, Lazartigues E (2020). ACE2 mouse models: a toolbox for cardiovascular and pulmonary research. Nat. Commun..

[26] Crawford, K. H. D. et al. Protocol and reagents for pseudotyping lentiviral particles with SARS-CoV-2 spike protein for neutralization assays. *Viruses*10.3390/v12050513 (2020). 10.3390/v12050513 PMC7291041 32384820

[27] Prieto-Fernandez E (2021). Hypoxia reduces cell attachment of SARS-CoV-2 spike protein by modulating the expression of ACE2, neuropilin-1, syndecan-1 and cellular heparan sulfate. Emerg. Microbes Infect..

[28] Harcourt J (2020). Severe acute respiratory syndrome coronavirus 2 from patient with coronavirus disease, United States. Emerg. Infect. Dis..

[29] Benedicto A, Garcia-Kamiruaga I, Arteta B (2021). Neuropilin-1: a feasible link between liver pathologies and COVID-19. World J. Gastroenterol..

[30] Stukalov A (2021). Multilevel proteomics reveals host perturbations by SARS-CoV-2 and SARS-CoV. Nature.

[31] Taneri PE (2020). Anemia and iron metabolism in COVID-19: a systematic review and meta-analysis. Eur. J. Epidemiol..

[32] Sonnweber T (2020). Persisting alterations of iron homeostasis in COVID-19 are associated with non-resolving lung pathologies and poor patients’ performance: a prospective observational cohort study. Respir. Res.

[33] Maio N (2021). Fe-S cofactors in the SARS-CoV-2 RNA-dependent RNA polymerase are potential antiviral targets. Science.

[34] Wenzhong L, Hualan L (2021). COVID-19: captures iron and generates reactive oxygen species to damage the human immune system. Autoimmunity.

[35] Del Nonno, F. et al. Hepatic failure in COVID-19: is iron overload the dangerous trigger? *Cells*10.3390/cells10051103 (2021). 10.3390/cells10051103 PMC8147922 34064487

[36] Walter PB (2002). Iron deficiency and iron excess damage mitochondria and mitochondrial DNA in rats. Proc. Natl Acad. Sci. USA.

[37] Galaris D, Barbouti A, Pantopoulos K (2019). Iron homeostasis and oxidative stress: an intimate relationship. Biochim. Biophys. Acta. Mol. Cell Res..

[38] Cao X, Song LN, Yang JK (2021). ACE2 and energy metabolism: the connection between COVID-19 and chronic metabolic disorders. Clin. Sci..

[39] Song LN (2020). Angiotensin-(1-7), the product of ACE2 ameliorates NAFLD by acting through its receptor Mas to regulate hepatic mitochondrial function and glycolipid metabolism. FASEB J..

[40] Shi TT (2018). Angiotensin-converting enzyme 2 regulates mitochondrial function in pancreatic beta-cells. Biochem. Biophys. Res. Commun..

[41] Focusing on mitochondrial form and function. *Nat. Cell Biol*. 10.1038/s41556-018-0139-7 (2018). 10.1038/s41556-018-0139-7 29950569

[42] Ott M, Gogvadze V, Orrenius S, Zhivotovsky B (2007). Mitochondria, oxidative stress and cell death. Apoptosis.

[43] Santos RAS (2018). The ACE2/angiotensin-(1-7)/MAS axis of the renin-angiotensin system: focus on angiotensin-(1-7). Physiol. Rev..

[44] Trougakos IP (2021). Insights to SARS-CoV-2 life cycle, pathophysiology, and rationalized treatments that target COVID-19 clinical complications. J. Biomed. Sci..

[45] Stefan N, Birkenfeld AL, Schulze MB (2021). Global pandemics interconnected - obesity, impaired metabolic health and COVID-19. Nat. Rev. Endocrinol..

[46] Simon J (2021). Magnesium accumulation upon cyclin M4 silencing activates microsomal triglyceride transfer protein improving NASH. J. Hepatol..

[47] Cao S (2010). Neuropilin-1 promotes cirrhosis of the rodent and human liver by enhancing PDGF/TGF-beta signaling in hepatic stellate cells. J. Clin. Invest..

[48] Troeger JS, Schwabe RF (2011). Neuropilin and liver fibrosis: hitting three birds with one stone. Hepatology.

[49] Li Y, Liu L, Wang B, Wang J, Chen D (2013). Metformin in non-alcoholic fatty liver disease: a systematic review and meta-analysis. Biomed. Rep..

[50] Rena G, Hardie DG, Pearson ER (2017). The mechanisms of action of metformin. Diabetologia.

[51] Pernicova I, Korbonits M (2014). Metformin-mode of action and clinical implications for diabetes and cancer. Nat. Rev. Endocrinol..

[52] Borem LMA, Neto JFR, Brandi IV, Lelis DF, Santos SHS (2018). The role of the angiotensin II type I receptor blocker telmisartan in the treatment of non-alcoholic fatty liver disease: a brief review. Hypertens. Res..

[53] Hegyi PJ (2021). Metabolic associated fatty liver disease is associated with an increased risk of severe COVID-19: a systematic review with meta-analysis. Front. Med..

[54] Letko M, Marzi A, Munster V (2020). Functional assessment of cell entry and receptor usage for SARS-CoV-2 and other lineage B betacoronaviruses. Nat. Microbiol..

[55] Chu H (2020). Multicenter analysis of liver injury patterns and mortality in COVID-19. Front. Med..

[56] Harcourt, J. et al. Isolation and characterization of SARS-CoV-2 from the first US COVID-19 patient. Preprint at *bioRxiv*10.1101/2020.03.02.972935 (2020).

[57] Yang L (2020). A human pluripotent stem cell-based platform to study SARS-CoV-2 tropism and model virus infection in human cells and organoids. Cell Stem Cell.

[58] McCarron S (2021). Functional characterization of organoids derived from irreversibly damaged liver of patients with NASH. Hepatology.

[59] Zhao B (2020). Recapitulation of SARS-CoV-2 infection and cholangiocyte damage with human liver ductal organoids. Protein Cell.

[60] Saleh J, Peyssonnaux C, Singh KK, Edeas M (2020). Mitochondria and microbiota dysfunction in COVID-19 pathogenesis. Mitochondrion.

[61] Icard P (2021). The key role of Warburg effect in SARS-CoV-2 replication and associated inflammatory response. Biochimie.

[62] Yaneske E, Zampieri G, Bertoldi L, Benvenuto G, Angione C (2021). Genome-scale metabolic modelling of SARS-CoV-2 in cancer cells reveals an increased shift to glycolytic energy production. FEBS Lett..

[63] Bharadwaj S, Singh M, Kirtipal N, Kang SG (2020). SARS-CoV-2 and glutamine: SARS-CoV-2 triggered pathogenesis via metabolic reprograming of glutamine in host cells. Front. Mol. Biosci..

[64] Simon J (2020). Targeting hepatic glutaminase 1 ameliorates non-alcoholic steatohepatitis by restoring very-low-density lipoprotein triglyceride assembly. Cell Metab..

[65] Wang Y (2019). Metformin improves mitochondrial respiratory activity through activation of AMPK. Cell Rep..

[66] Han Y (2019). Post-translational regulation of lipogenesis via AMPK-dependent phosphorylation of insulin-induced gene. Nat. Commun..

[67] Song L (2018). LKB1 obliterates Snail stability and inhibits pancreatic cancer metastasis in response to metformin treatment. Cancer Sci..

[68] Tolosa L (2016). Human upcyte hepatocytes: characterization of the hepatic phenotype and evaluation for acute and long-term hepatotoxicity routine testing. Toxicol. Sci..

[69] Hughes CS (2019). Single-pot, solid-phase-enhanced sample preparation for proteomics experiments. Nat. Protoc..

[70] Meier F (2015). Parallel accumulation-serial fragmentation (PASEF): multiplying sequencing speed and sensitivity by synchronized scans in a trapped ion mobility device. J. Proteome Res..

[71] Tyanova S (2016). The Perseus computational platform for comprehensive analysis of (prote)omics data. Nat. Methods.

[72] Fernandez-Tussy P (2021). Anti-miR-518d-5p overcomes liver tumor cell death resistance through mitochondrial activity. Cell Death Dis..

[73] Dieterle F, Ross A, Schlotterbeck G, Senn H (2006). Probabilistic quotient normalization as robust method to account for dilution of complex biological mixtures. Application in 1H NMR metabonomics. Anal. Chem..

[74] Veselkov KA (2011). Optimized preprocessing of ultra-performance liquid chromatography/mass spectrometry urinary metabolic profiles for improved information recovery. Anal. Chem..

