# Supplementary material for: The spike of SARS-CoV-2 promotes metabolic rewiring in hepatocytes
Source: Commun Biol. 2022 Aug 17;5:827. doi: 10.1038/s42003-022-03789-9 (PMC9383691; doi:10.1038/s42003-022-03789-9)
Supplement: Supplementary file 2 — Supplementary Information [file 42003_2022_3789_MOESM2_ESM.pdf]

SUPPLEMENTARY INFORMATION

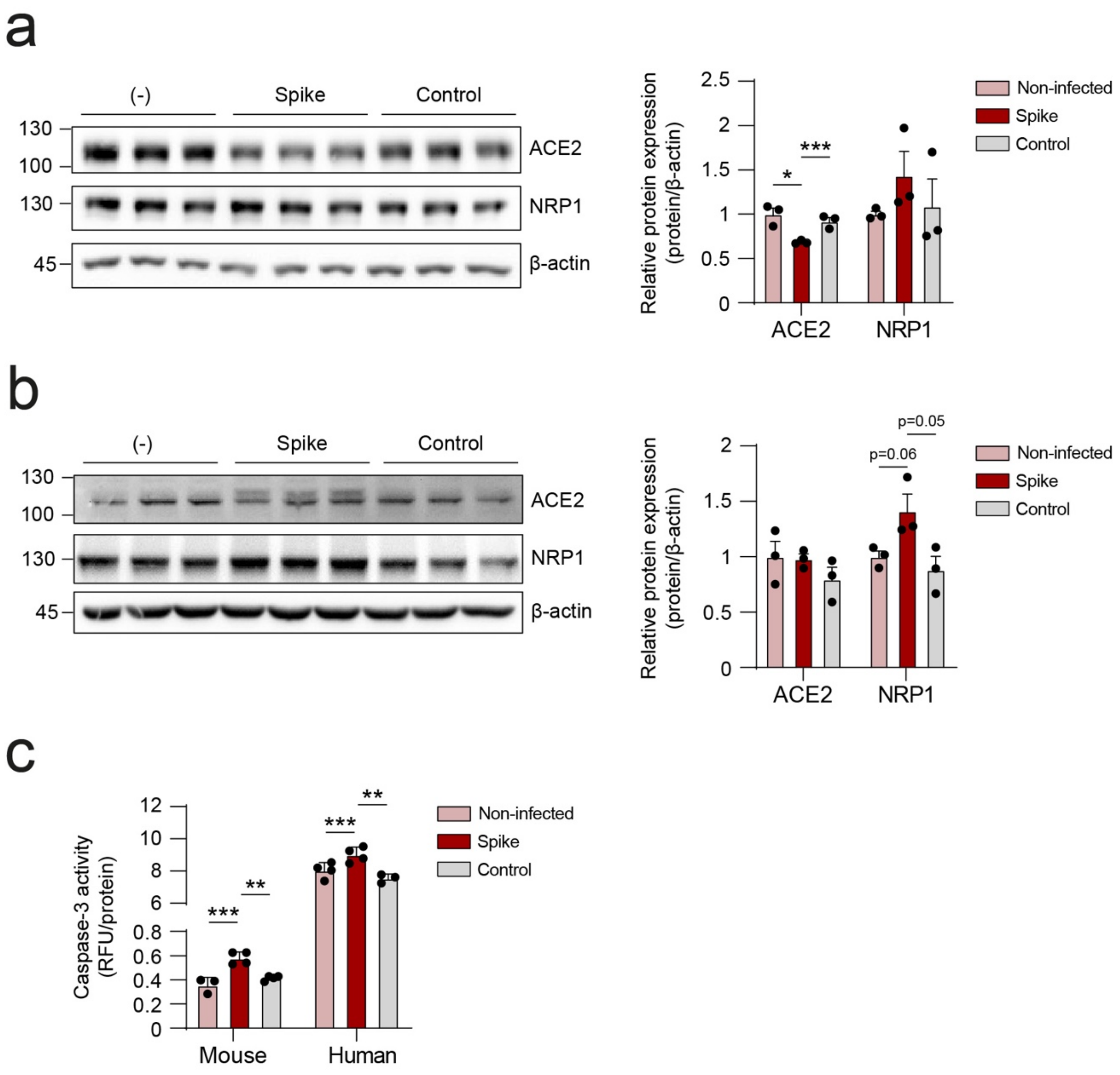

Supplementary Fig. 1. Binding of pseudotyped viral particles expressing the SARS-CoV-2 spike alters the expression of ACE2 and NRP1 proteins and induces apoptosis in hepatocytes. (**a, b**) Western blot showing the expression of ACE2 and NRP1 proteins on primary hACE2 mouse hepatocytes (*a*) or upcyte second-generation human hepatocytes (*b*) infected with lentiviral particles expressing the spike protein or control (n=3, one-way ANOVA test). (**c**) Caspase-3 activity measured by fluorimetry in mouse and human primary hepatocytes treated with lentiviral particles expressing the spike protein or control (n=4, one-way ANOVA test). Error bars represent SEM and asterisks represent p-values (*<0.05, **<0.01, ***<0.001).

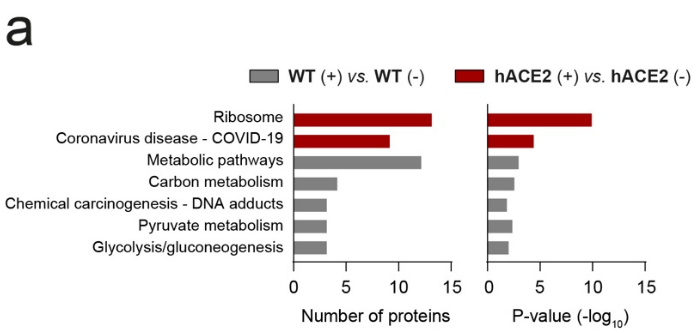

Supplementary Fig 2. Infection with pseudotyped viral particles alters ribosome- and COVID-19-related processes on hACE2 hepatocytes compared to control hepatocytes. (**a**) KEGG pathways representing the unique differentially expressed peptides in WT or hACE2 mouse hepatocytes either treated with pseudotyped lentiviral particles expressing the spike or SARS-CoV-2 (+) or control viral particles (-). Number of proteins belonging the identified dysregulated pathways (left) and their corresponding p-values (right) are shown.

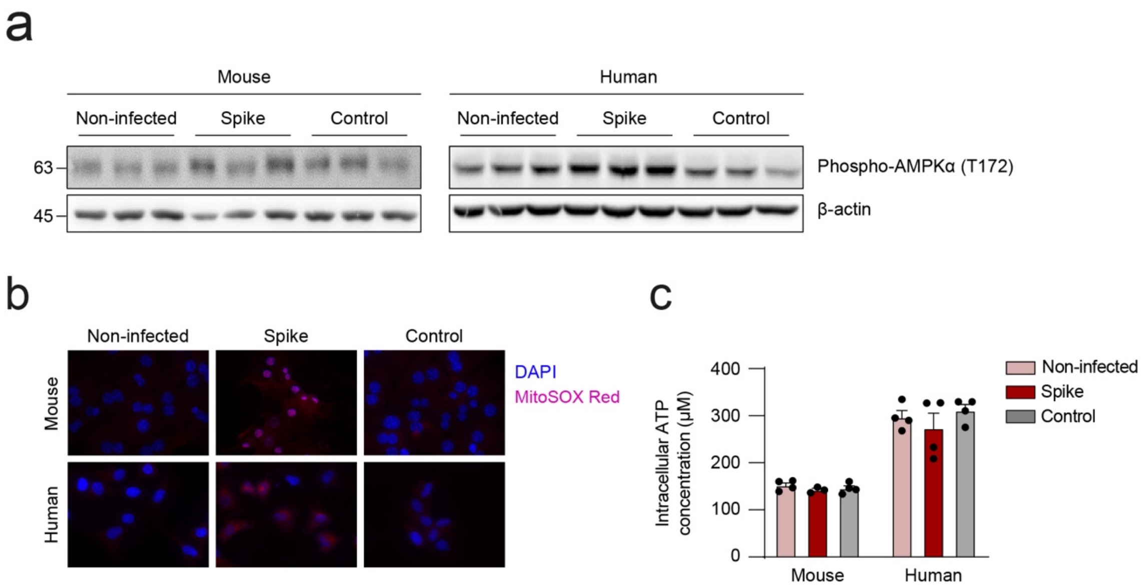

Supplementary Fig. 3. Binding of the spike of SARS-CoV-2 alters mitochondrial activity and glucose homeostasis in hACE2 and human primary hepatocytes. (**a**) Western blot showing the expression of phospho-AMPKα (T172) protein on hACE2 (*left*) and primary human hepatocytes (*right*) infected with lentiviral particles expressing the spike protein or control. (**b**) Representative images of hACE2 and human hepatocytes labelled with MitoSOX for the indicated conditions. (**c**) Intracellular ATP concentration in both mouse (hACE2) and human primary hepatocytes after infection with the indicated lentiviral particles (n= 4, one-way ANOVA).

a

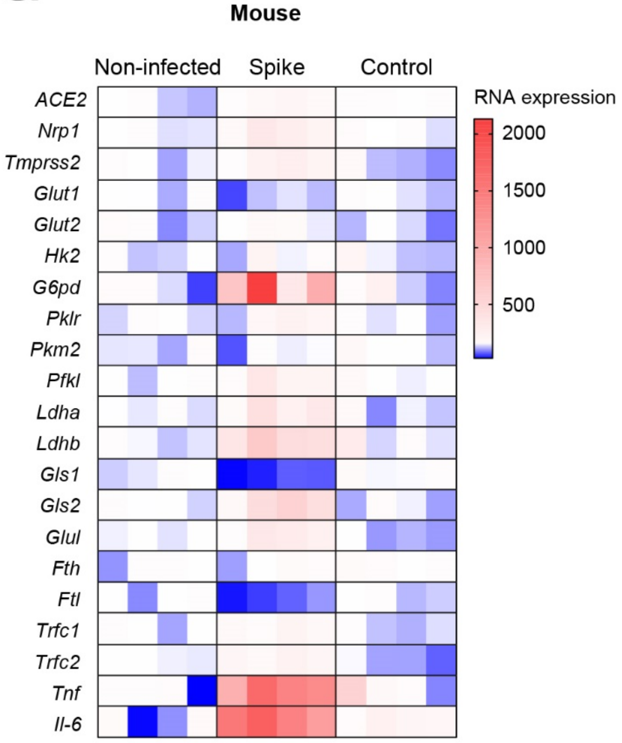

	Non-infected *vs.* Spike	Control *vs.* Spike	Non-infected *vs.* Control
ACE2	*	*	ns
Nrp1	**	*	ns
Tmprss2	*	*	ns
Glut1	ns	ns	ns
Glut2	ns	ns	ns
Hk2	ns	ns	ns
G6pd	p=0.05	ns	ns
Pklr	p=0.07	p=0.06	ns
Pkm2	ns	ns	ns
Pfkl	*	p=0.06	ns
Ldha	*	*	ns
Ldhb	*	**	ns
Gls1	***	***	ns
Gls2	*	*	ns
Glul	*	*	ns
Fth	ns	ns	ns
Ftl	*	*	ns
Trfc1	**	**	ns
Trfc2	**	***	ns
Tnf	***	***	ns
Il-6	ns	ns	ns

b

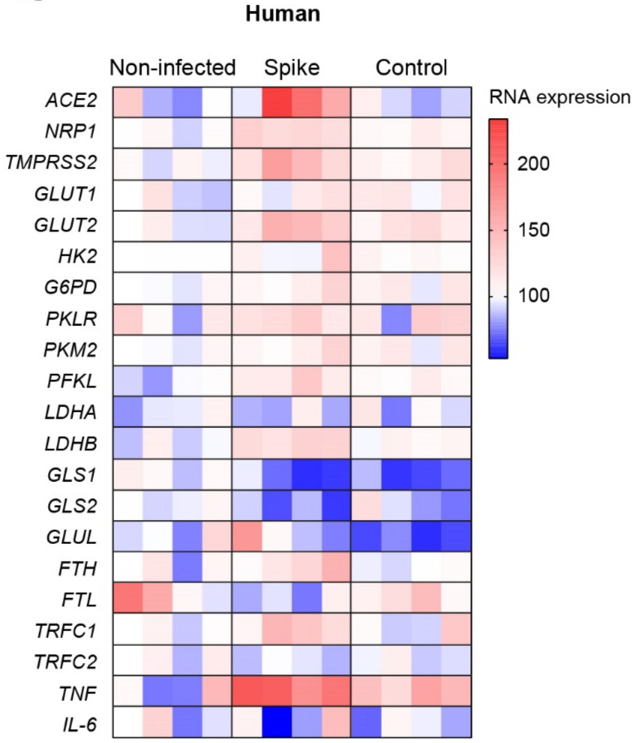

	Non-infected *vs.* Spike	Control *vs.* Spike	Non-infected *vs.* Control
ACE2	p=0.06	ns	ns
NRP1	***	***	ns
TMPRSS2	**	*	ns
GLUT1	ns	ns	ns
GLUT2	*	ns	ns
HK2	ns	ns	ns
G6PD	p=0.06	ns	ns
PKLR	p=0.06	ns	ns
PKM2	ns	ns	ns
PFKL	ns	ns	ns
LDHA	ns	ns	ns
LDHB	**	**	**
GLS1	**	ns	**
GLS2	*	ns	ns
GLUL	*	ns	*
FTH	ns	*	ns
FTL	ns	ns	ns
TRFC1	*	ns	ns
TRFC2	ns	ns	ns
TNF	**	*	ns
IL-6	ns	ns	ns

Supplementary Fig. 4. Binding of the spike of SARS-CoV-2 alters mitochondrial activity and glucose homeostasis in hACE2 and human primary hepatocytes. (**a, b**) RNA expression of genes related to SARS-CoV-2 infection and inflammatory

responses, as well as other related with glucose, lactate and glutamine metabolism on mouse (*a*) and human (*b*) hepatocytes after infection with pseudotyped viral particles expressing the spike of SARS-CoV-2 or controls (n=4, one-way ANOVA). Asterisks represent p-values (*<0.05, **<0.01 and ***<0.001).

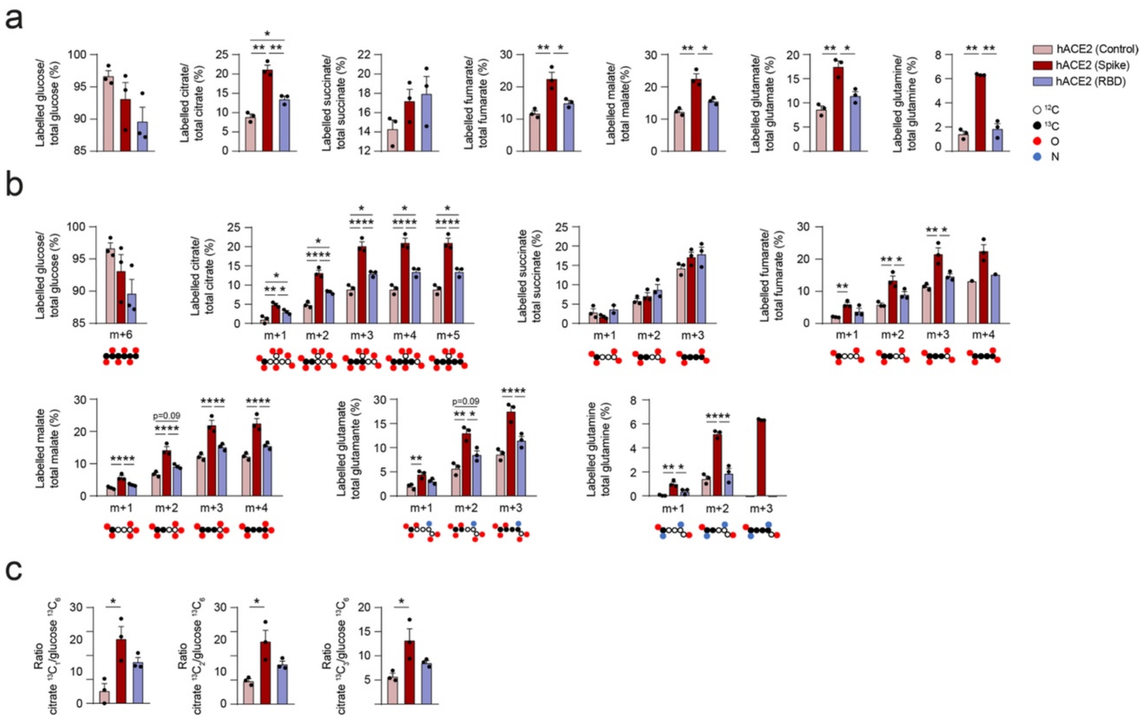

Supplementary Fig. 5. Binding of the spike of SARS-CoV-2 alters mitochondrial activity and glucose homeostasis in hACE2 and human primary hepatocytes. (**a**) Percentage of total labelled carbons of each metabolite (glucose, citrate, succinate, fumarate, malate, glutamate and glutamine) related to their total levels measured on hACE2 hepatocytes exposed to pseudotyped viral particles or recombinant RBD (n=3, one-way ANOVA). (**b**) Percentage of every labelled carbon (^{13}C) of the metabolites analyzed related to their total amount (n= 3, one-way ANOVA). (**c**) Ratios between 1/2/3-^{13}C (citrate) related to 6-^{13}C (glucose) (n= 3, one-way ANOVA). Abbreviations: α-KG: α-ketoglutarate; ^{12}C: carbon-12 molecule; ^{13}C: carbon-13 isotope; O: oxygen molecule; N: nitrogen molecule. Asterisks represent p values (*<0.05 and **<0.01).

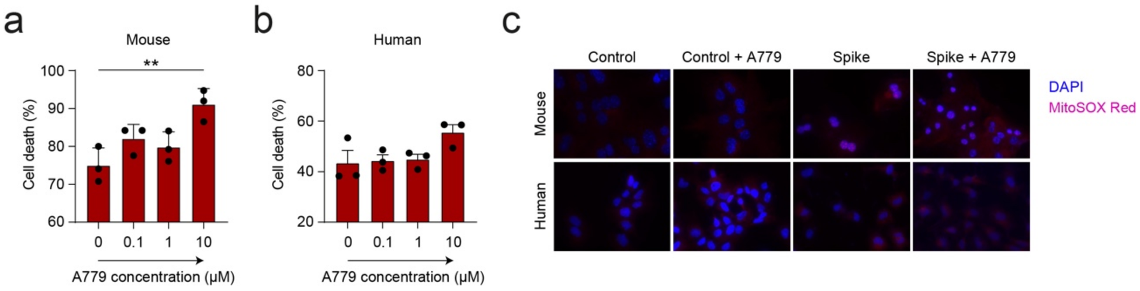

Supplementary Fig. 6. Infection of primary hepatocytes regulates the renin-angiotensin system. (**a, b**) Percentage of cell death corresponding to hACE2 (*a*) and human (*b*) primary hepatocytes after infection with pseudotyped lentiviral particles expressing the spike of SARS-CoV-2 in the presence of the indicated doses of A779 or vehicle control, measured by flow cytometry (n=3, one-way ANOVA). (**c**) Representative images of hACE2 and human hepatocytes labelled with MitoSOX after infection with pseudotyped viral particles or controls in the presence or absence of A779 (mouse: 0.1 µM; human: 1µM). Error bars represent SEM and asterisks represent p-values (**<0.01).

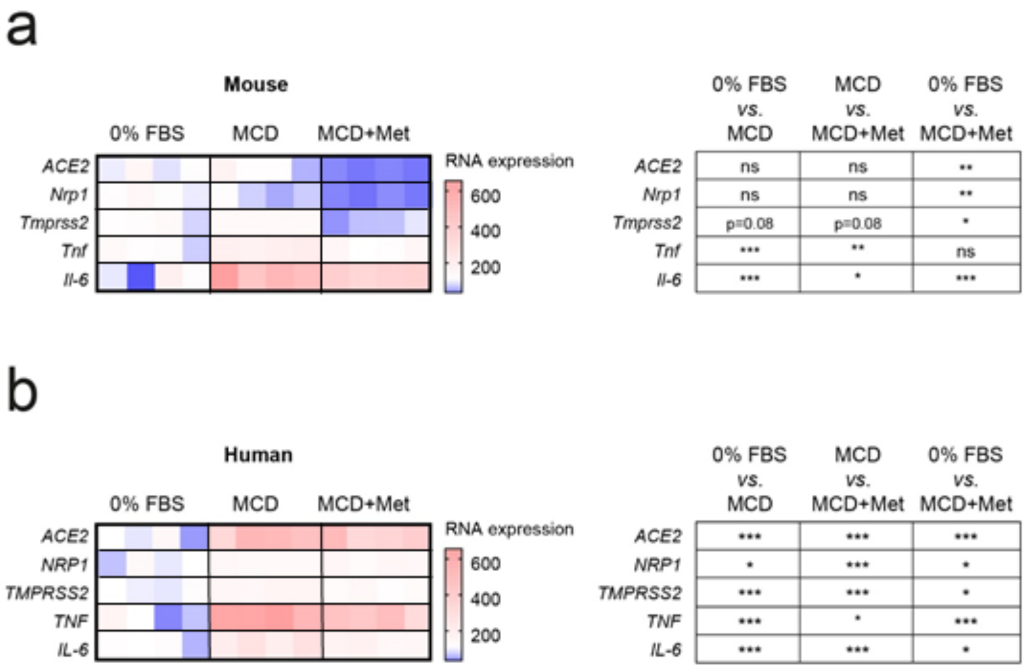

Supplementary Fig. 7. Dysregulation of hepatocyte mitochondrial activity modulates ACE2 levels and increases susceptibility to infection. (**a, b**) RNA expression of the indicated genes on mouse (*a*) and human (*b*) hepatocytes treated with MCD or MCD+Metormin (Met) compared to controls (0% FBS). Asterisks represent p-values (*<0.05, **<0.01 and ***<0.001).

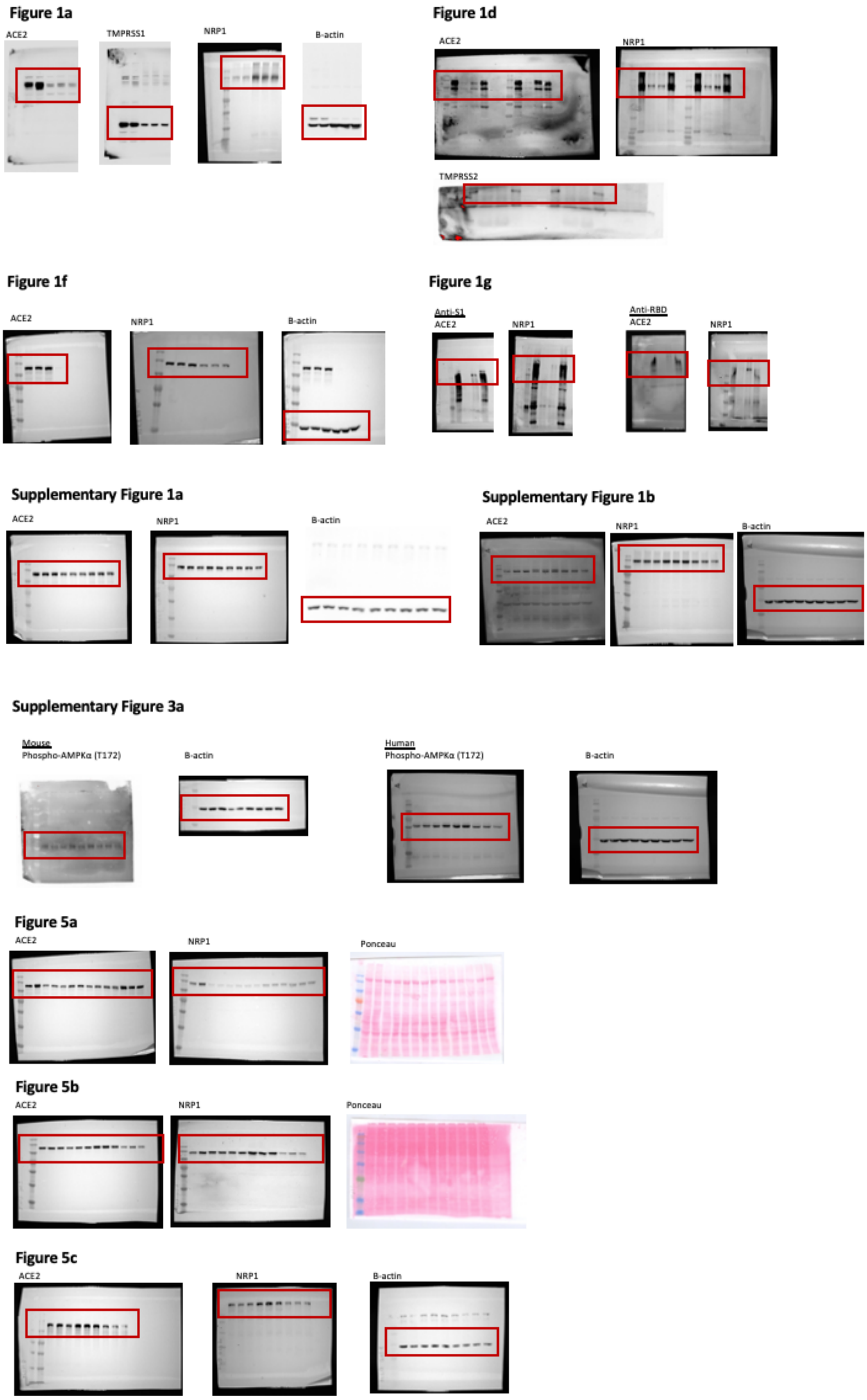

Supplementary Fig. 8. Uncropped and unedited blot images.
