# Supplementary material for: The spike of SARS-CoV-2 promotes metabolic rewiring in hepatocytes
Source: Commun Biol. 2022 Aug 17;5:827. doi: 10.1038/s42003-022-03789-9 (PMC9383691; doi:10.1038/s42003-022-03789-9)
Supplement: Supplementary file 3 — Description of Additional Supplementary Files [file 42003_2022_3789_MOESM3_ESM.pdf]

Description of Additional Supplementary Files

File name: Supplementary Data 1
Description: A detailed list of all peptides detected by mass spectrometry-based proteomics and the fold-change and p-values for the indicated groups.

File name: Supplementary Data 2
Description: : List of primers used for mRNA quantification by quantitative PCR.

File name: Supplementary Data 3
Description: Source data underlying the graphs and charts presented in the manuscript.
